# Delineation of immunodominant and cytadherence segment(s) of *Mycoplasma pneumoniae* P1 gene

**DOI:** 10.1186/1471-2180-14-108

**Published:** 2014-04-28

**Authors:** Bishwanath K Chourasia, Rama Chaudhry, Pawan Malhotra

**Affiliations:** 1 Department of Microbiology, All India Institute of Medical Sciences, New Delhi, India; 2 International Centre for Genetic Engineering and Biotechnology, New Delhi, India

## Abstract

**Background:**

Adhesion of *Mycoplasma pneumoniae* (*M. pneumoniae*) to host epithelial cells requires several adhesin proteins like P1, P30 and P116. Among these proteins, P1 protein has been inedited as one of the major adhesin and immunogenic protein present on the attachment organelle of *M. pneumoniae*. In the present study, we scanned the entire sequence of *M. pneumoniae* P1 protein to identify the immunodominant and cytadherence region(s). *M. pneumoniae* P1 gene was synthesized in four segments replacing all the UGA codons to UGG codons. Each of the four purified P1 protein fragment was analyzed for its immunogenicity with anti-*M. pneumoniae* M129 antibodies (Pab M129) and sera of *M. pneumoniae* infected patients by western blotting and ELISA. Antibodies were produced against all the P1 protein fragments and these antibodies were used for *M. pneumoniae* adhesion, *M. pneumoniae* adhesion inhibition and *M. pneumoniae* surface exposure assays using HEp-2 cells lines.

**Results:**

Our results show that the immunodominant regions are distributed throughout the entire length of P1 protein, while only the N- and C- terminal region(s) of P1 protein are surface exposed and block cytadhesion to HEp-2 cells, while antibodies to two middle fragments failed to block cytadhesion.

**Conclusions:**

These results have important implications in designing strategies to block the attachment of *M. pneumoniae* to epithelial cells, thus preventing the development of atypical pneumonia.

## Background

M*ycoplasmas* are the smallest known self-replicating prokaryotes originally isolated from bovine pleuropneumonia and are also referred as pleuropneumonia like organisms (PPLO). A key characteristic of mycoplasma is the lack of a cell wall, which allows exchange of different components between the host membrane and the *M. pneumoniae* membrane after adhesion [1,2]. *M. pneumoniae* is a human pathogen that colonizes the ciliated upper and lower respiratory tract, causing atypical pneumonia. *M. pneumoniae* is also found to be associated with other respiratory tract infections such as tracheobronchitis, bronchiolitis, croup, Acute Respiratory Distress Syndrome (ARDS), Guillain-Barre Syndrome (GBS), stroke and less severe upper respiratory tract infections in older children as well as in young adults [3-7]. Adherence of *M. pneumoniae* to the human host respiratory epithelium is a prerequisite for the colonization and subsequent induction of disease [4,8]. It attaches to ciliated epithelial cells in the respiratory tract, where it induces ciliostasis that protects the *M. pneumoniae* from removal by the mucociliary clearance mechanism of the host [9]. *M. pneumoniae* is elongated and consists of a longer tail-like rear end, a thicker body part and a frontal attachment organelle.

Cytadherence requires a complex interaction of several *M. pneumoniae* proteins present on the attachment organelle, including the adhesins P1 (170 kDa), P30 (30 kDa), and P116 (116 kDa) and proteins HMW1 to HMW3, as well as proteins A, B and C [4,10-15]. Protein P1 and P30 appear to be directly involved in receptor binding [8,16]. The HMW proteins and proteins A, B, and C are accessory proteins as they are not adhesins, but are required for proper attachment. The P1 protein, which is mainly concentrated at the tip of apical organelle, is one of the major adhesins in *M. pneumoniae* as mutants lacking the P1 protein lose cytadherence and virulence capabilities [17,18]. In addition, treatment of *M. pneumoniae* infection with anti-P1 antibodies has been shown to effect the gliding speed of *M. pneumoniae*, thus hampering the mobility of the bacterium and possibly its ability to find suitable host adhesion receptors [19]. Besides its role in *M. pneumoniae* cytadherence, P1 antigen is an important immunogen and is also being developed as defined and specific antigen for the serodiagnosis of *M. pneumoniae* infection [20]. Previous reports and we have shown that a C-terminal region of P1 antigen can comparably diagnose *M. pneumoniae* infection taking the Serion-Virion ELISA as the standard [14,21]. Serum samples from patients suffering from *M. pneumoniae* infection have also been shown to bind the peptide fragments located in the middle of the ~170 kDa P1 antigens [22].

Since P1 is one of the major surface molecules on the apical organelles of *M. pneumoniae*, a number of studies have been performed to determine its immunogenicity as well as to characterize its role in adhesion/cytadherence. Using λgt11 recombinant DNA expression library of *M. pneumoniae,* Dallo *et al.* for the first time identified cytadherence (epitopes) at the C-terminal region of P1 gene [23]. Subsequently, in two independent studies based on topological mapping of the P1 binding sites, Gerstenecker *et al.* and Opitz *et al.* identified adherence associated region(s) across the length of P1 gene [11,24]. Jacobs *et al.* further defined immunodominant epitopes of 338 amino acids between leucine 801 and leucine 1139 residues [25]. In 2002, Svenstrup *et al.* expressed P1 fragments lacking the tryptophan codon which codes for a stop codon in *M. pneumoniae* and identified adhesion epitopes in the C-terminal part of *M. pneumoniae* P1 gene using monospecific antibodies [14].

Although these above mentioned studies identified few adhesion/cytadherence segment(s) in *M. pneumoniae* P1 protein, a systematic study defining the region(s) involved in these processes across the entire length of P1 protein is lacking, therefore leading to contradicting results. One of the main reasons for the lack of a systematic study is the presence of 21 UGA codons, which makes it difficult to express this protein or its fragments in heterologus systems such as *Escherichia coli* or mammalian systems. To circumvent this problem, PCR-based site-directed mutagenesis may have been one of method to replace TGA codons in P1 gene as mentioned by Hames *et al.*[26], but we decided to synthesize the entire P1 gene into four different fragments by codon optimization. This included the N-terminal (P1-I) fragment, two middle fragments P1-II and P1-III and a C-terminal (P1-IV) fragment, which have been suggested to be immunodominant and to act as adhesins [14,21,25,27]. All these fragments were cloned and expressed in an *E. coli* system [28-30]. The immunological and cytadherence characterization of all the four P1 protein fragments identified specific cytadherence regions. These results will enable to define strategies for the development of drug/vaccine against *M. pneumoniae* infection.

## Results

### Cloning, expression and purification of P1 gene fragments

**Figure 1:**
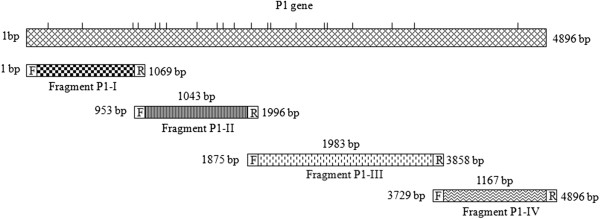
**Schematic representation of *M. pneumoniae *M129 P1 gene and its four gene fragments; P1-I, P1-II, P1-III and P1-IV.** Each bar represents the position of UGA codons that codes for tryptophan. To express these fragments, UGA codons were modified to UGG. Fragments were amplified using a set of forward (F) and reverse primers (R).

**Figure 2:**
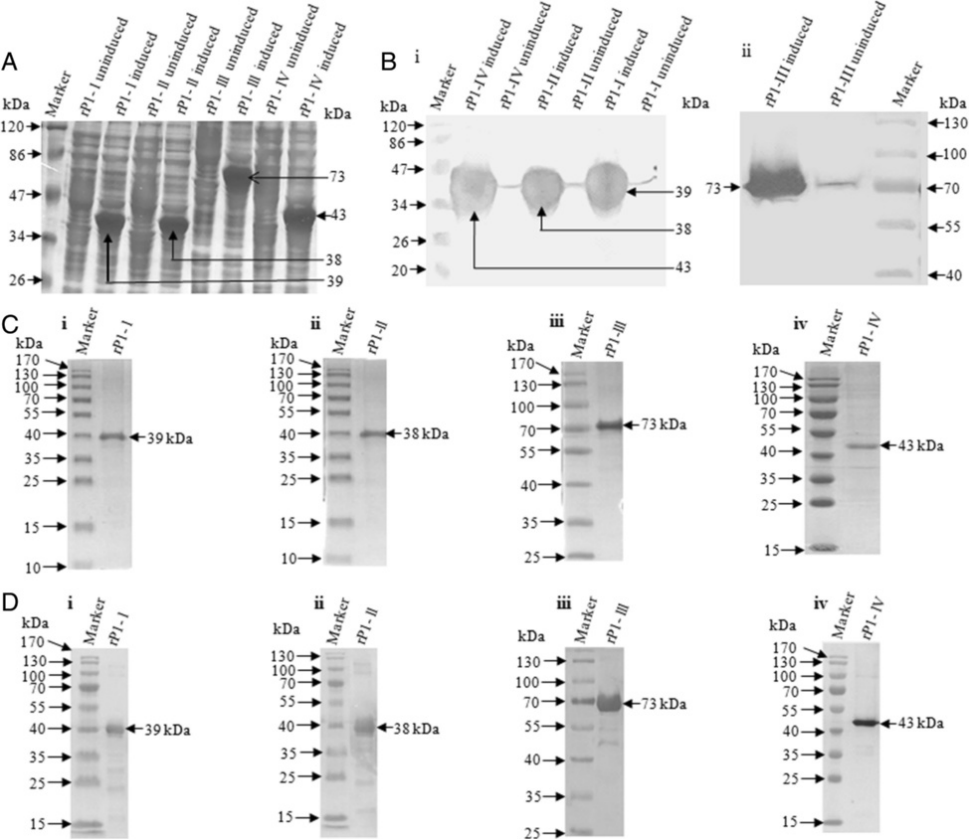
**SDS-PAGE and Western blot analysis of recombinant *M. pneumoniae *P1 proteins fragments. (A)** Coomassie blue stained SDS-PAGE analysis of rP1-I, rP1-II, rP1-III and rP1-IV in *E. coli* extract. The fragments were expressed in pET28b vector and protein production was induced with IPTG in *E. coli.***(B)** Western blot analysis of induced and uninduced P1 protein fragments rP1-I, rP1-II, rP1-IV (i) and rP1-III (ii), showing reactivity with anti-6X His antibody. **(C)** Coomassie blue stained SDS-PAGE analysis of Ni^2+^-NTA purified P1 protein fragments; rP1-I, rP1-II, rP1-III and rP1-IV. **(D)** Western blot analysis of purified P1 protein fragments rP1-I, rP1-II, rP1-III and rP1-IV showing reactivity with anti-6X His antibody. Lane Marker: Molecular mass marker (kDa); Arrows indicate position of expressed protein.

Four fragments of the *M. pneumoniae* P1 gene, i.e., P1-I, P1-II, P1-III, & P1-IV (Figure 1), were amplified by PCR, cloned in expression vector pET28b and expressed in *E. coli* BL21(DE3) cells. The expressed proteins were analyzed on SDS-PAGE. As shown in Figure 2A, four proteins of molecular weights: ~39 kDa, ~38 kDa, ~73 kDa, and ~43 kDa were induced and they were mainly expressed in inclusion bodies. The expressions of recombinant proteins were further confirmed by western blot analysis using anti-6XHis antibody (Figure 2B i & ii). The expressed proteins were purified up to near homogeneity on a Ni^2+^-NTA column (Figure 2C). Fractions that contained single band for each of the recombinant protein were pooled, dialyzed and further characterized. The expressed and purified proteins reacted nicely with anti-6XHis antibody (Figure 2D).

### Recombinant rP1-I, rP1-II, rP1-III and rP1-IV proteins are immunogenic

**Figure 3:**
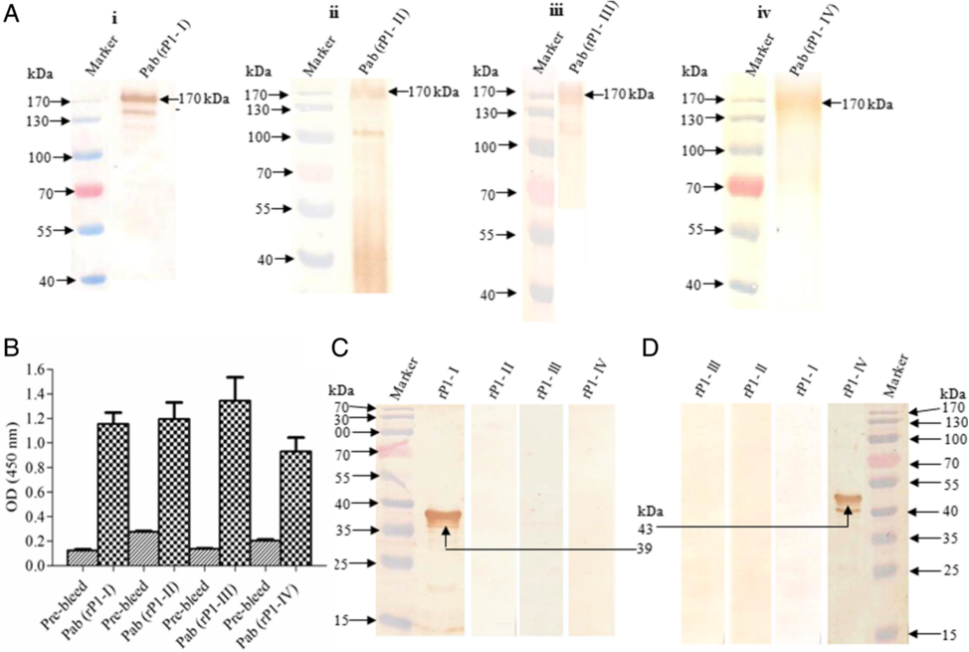
**Western blot and ELISA analysis of *M. pneumoniae *lysate and Cross reactivity of Pab (rP1-I) and Pab (rP1-IV).** Reactivity of P1 (170 kDa) with anti-P1 protein fragments antibody Pab (rP1-I), Pab (rP1-II), Pab (rP1-III) & Pab (rP1-IV) rose in Rabbit by western blotting **(A)** and by ELISA **(B)**. **(C)** &**(D)** Immuno blot analysis of rP1-I, rP1-II, rP1-III and rP1-IV fragments with Pab (rP1-I) and Pab (rP1-IV) showing their cross reactivity with respective sera. Lane Marker: Molecular mass marker (kDa).

High antibody responses were seen against each of the four recombinant proteins. The time course response for each of the recombinant proteins showed that the antibody titers gradually increased after first and second booster and peaked after the second boost. An additional figure file [see Additional file 1] shows the time dependent response for recombinant P1-I protein. Almost similar antigenic responses were observed for other three P1 protein fragments (data not shown). The end point titers for each protein were > 1 × 10^5^. Western blotting for all the four recombinant proteins with their respective antibodies confirmed the specificity of each antibody. All these antibodies showed major reactivity with ~170 kDa band of P1 protein in *M. pneumoniae* lysate by ELISA (Figure 3B) and western blotting. Anti-P1 antibodies also reacted with few additional bands in *M. pneumoniae* lysate. These additional bands probably represent the degraded P1 protein bands (Figure 3A). No cross reactivity was observed between each of the four antibodies (Figure 3C &3D). Almost similar reactivity was observed with two other P1 protein fragments rP1-II & rP1-III (data not shown). These results indicated that all the four P1 protein fragments are immunogenic and antibodies are specific as they only recognized the corresponding protein fragment. Pre-bleed and control rabbit sera showed no reactivity with any of the recombinant protein fragments. An additional figure file [see Additional file 2] shows the reactivity of each protein fragment with pre-bleed sera.

### Recombinant rP1-I, rP1-II, rP1-III and rP1-IV proteins were recognized by anti-*M. pneumoniae* antibody and by sera of *M. pneumoniae* infected patients

**Figure 4:**
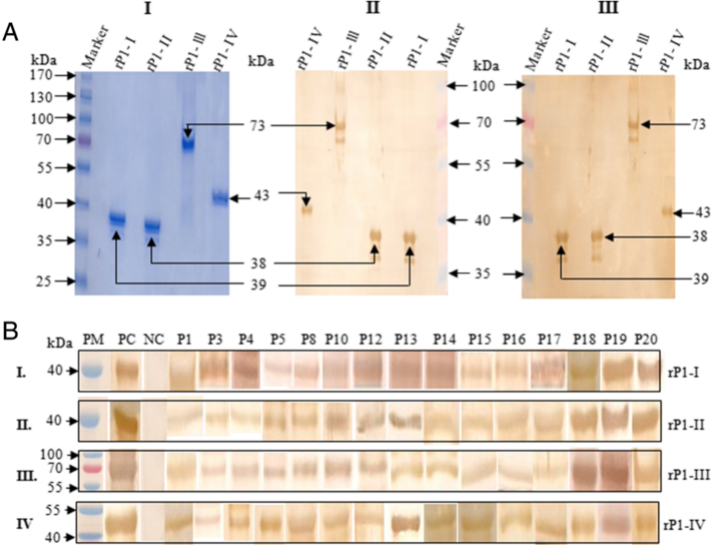
**Recombinant P1 protein fragments are recognized by anti-*M. pneumoniae *antibody and by sera of *M. pneumoniae *infected patients. (A)** (I) Coomassie blue stained SDS-PAGE analysis of purified *M. pneumoniae* P1 protein fragments; rP1-I, rP1-II, rP1-III and rP1-IV. Immuno blot analysis of purified P1 protein fragments; rP1-I, rP1-II, rP1-III and rP1-IV using anti-*M. pneumoniae* antibody (II) and using pooled sera of *M. pneumoniae* infected patients (III). **(B)** Immuno blot analysis of purified *M. pneumoniae* P1 protein fragments rP1-I, rP1-II, rP1-III and rP1-IV with several sera of *M. pneumoniae* infected patients. PM: Prestained protein marker; PC: positive control; NC: Negative control; Numbers over the blot indicate serial number of sera of *M. pneumoniae* infected patients tested for these experiments.

**Figure 5:**
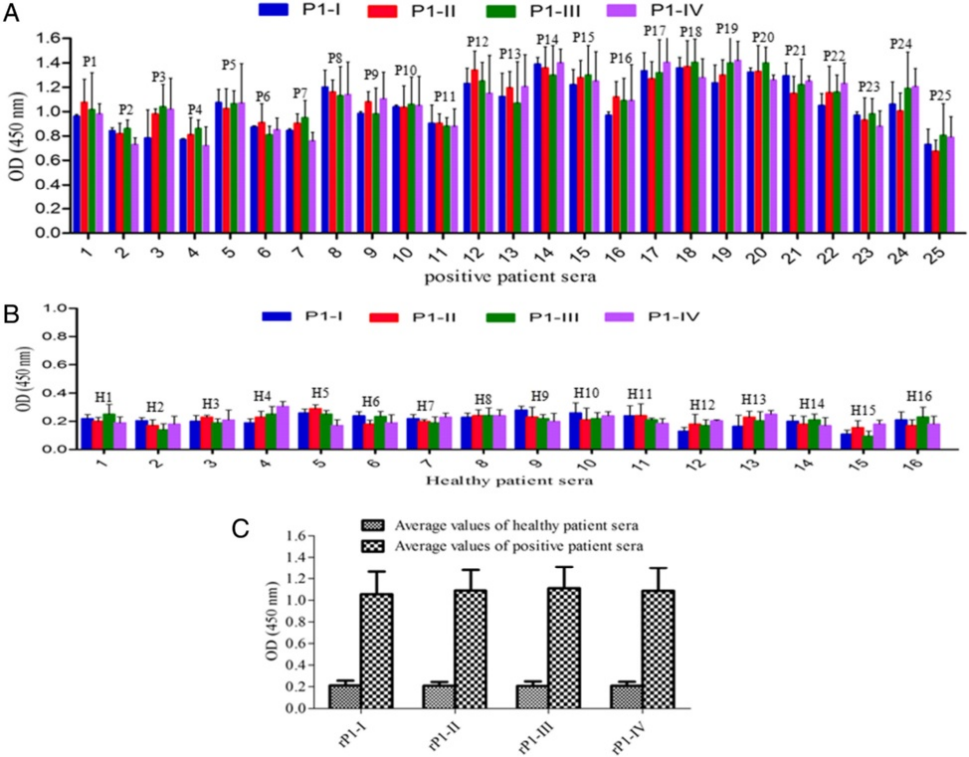
**Comparative ELISA analysis of recombinant P1 protein fragments with sera of *M. pneumoniae *infected patients.** Reactivity of purified *M. pneumoniae* P1 proteins fragments with 25 sera of *M. pneumoniae* infected patients by ELISA **(A)**, with 16 healthy patient sera **(B)** and average values of both **A** &**B (C)**. Number on top of column indicates serial number of sera of *M. pneumoniae* infected patients tested for these experiments.

All the four recombinant proteins were analyzed for their reactivity to anti-*M. pneumoniae* antibody and pooled sera of *M. pneumoniae* infected patients. To do so, 1 μg of each recombinant protein was loaded on SDS-PAGE gel (Figure 4A-I) and the proteins were blotted to nitrocellulose membrane. As shown in Figures 4A-II & III, all the four proteins showed similar reactivity with either of the two sera. We next compared the reactivity of the four recombinant proteins with fifteen and twenty-five sera of *M. pneumoniae* infected patients by western blot analysis and by ELISA respectively. Figures 4B &5A shows the reactivity of the recombinant proteins with sera of *M. pneumoniae* infected patients. Similar reactivity was seen for each of the four recombinant P1 protein fragments, thereby suggesting that the immunodominant regions are distributed across the entire length of P1 protein.

### *M. pneumoniae* adhesion and surface exposure assays reveal that P1-I and P1-IV regions are surface exposed.

**Figure 6:**
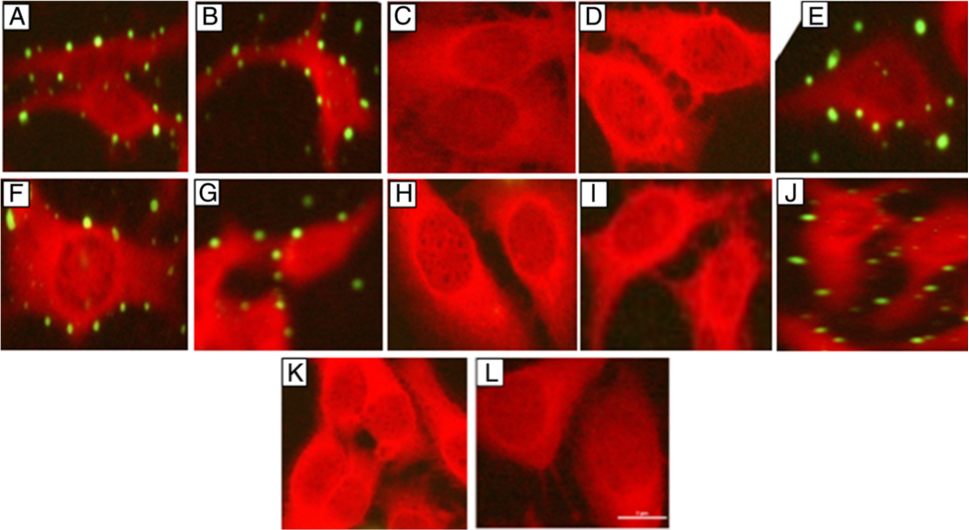
**IFM adhesion assay of *M. pneumoniae *(A-E).** The *M. pneumoniae* attached to the HEp-2 cells were detected by either anti-*M. pneumoniae* antibody or antibodies rose in rabbits. The detecting antibodies were added after fixation with methanol. **(A)** anti-*M. pneumoniae* antibody (positive control), **(B)** Pab (rP1-I), **(C)** Pab (rP1-II), **(D)** Pab (rP1-III), **(E)** Pab (rP1-IV). IFM surface exposure assay of *M. pneumoniae***(F-J)**. In this assay the detecting antibodies were added before the methanol fixation. **(F)** anti-*M. pneumoniae* antibody (positive control), **(G)** Pab (rP1-I), **(H)** Pab (rP1-II), **(I)** Pab (rP1-III), **(J)** Pab (rP1-IV). Negative controls: **(K)** mycoplasmas alone (Without Pabs), **(L)** Pabs alone (Without mycoplasmas). Bar, 2 μm.

For the adhesion assay, HEp-2 cells were infected with *M. pneumoniae* and methanol fixed before exposing them with each of the four anti-P1 antibodies; Pab (rP1-I), Pab (rP1-II), Pab (rP1-III), and Pab (rP1-IV) antibody. The bound antibodies were detected with an FITC-conjugated goat anti-rabbit immunoglobulin. As shown in Figure 6 (A-E), Indirect immunofluorescence microscopy analysis showed that the antibodies, Pab (rP1-I and Pab (rP1-IV were able to identify *M. pneumoniae* bound to the HEp-2 cells, while other two antibodies, Pab (rP1-II) and Pab (rP1-III) failed to identify the bound organism to HEp-2 cells.

To detect the accessibility of the antibodies on the surface of the cytadhering *M. pneumoniae*, the primary antibodies were added before fixation with methanol. The bound primary antibodies were detected with FITC-conjugated goat anti-rabbit IgG antibody followed by immunofluorescence microscopy. As seen in the case of adhesion detection assay, only the antibodies Pabs, rP1-I and rP1-IV were able to detect cytadhering *M. pneumoniae*, while no fluorescence was observed when antibodies Pabs, (rP1-II) and (rP1-III) were used (Figure 6 (F-J).

### *M. pneumoniae* adhesion inhibition assay

**Figure 7:**
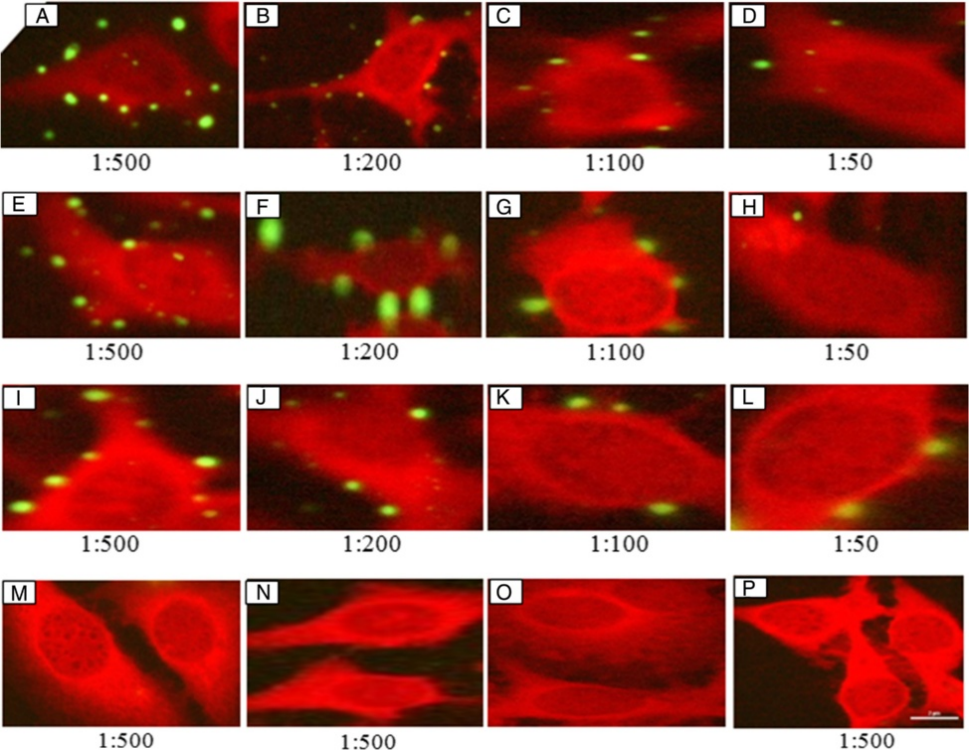
**IFM adhesion inhibition assay.***M. pneumoniae* were pre-incubated with either anti-*M. pneumoniae* antibodies or antibodies rose in rabbits in different dilutions (1:50, 1:100, 1:200, 1:500) before infection of the HEp-2 cells. These antibodies were: **(A-D)** anti-*M. pneumoniae* antibody (positive control), **(E-H)** Pab (rP1-I), **(I-L)** Pab (rP1-IV), **(M)** Pab (rP1-II) **(N)** Pab (rP1-III) **(O)** Without antibody, **(P)** pre-immune serum. Bar, 2 μm.

To examine the ability of each of the specific antibodies to block *M. pneumoniae* binding to HEp-2 cells, each of the four antibodies were diluted in four different concentrations 1:50, 1:100, 1:200 and 1:500 (200, 100, 50 and 20 μg/ml respectively). The diluted antibodies were incubated with the *M. pneumoniae* before infection with the HEp-2 cells. The *M. pneumoniae* attached to the HEp-2 cells were visualized by anti-*M. pneumoniae* sera and secondary FITC-conjugated goat anti-rabbit IgG antibody. Among these four specific antibodies, Pab (rP1-I) and Pab (rP1-IV) inhibited the adhesion of *M. pneumoniae* to the HEp-2 cells (Figures 7E-H & I-L). The inhibition was maximum at highest concentration of antibody (1:50) and inhibition decreased as concentration of antibodies decreased and almost no inhibition were seen with the minimum concentration of antibody (1:500 dilution). In an independent experiment, we also performed DAPI staining to confirm adhesion inhibition by Pab (rP1-I) and Pab (rP1-IV) antibodies [see Additional file 3]. Importantly, antibodies; Pab (rP1-II) and Pab (rP1-III) failed to block the *M. pneumoniae* adhesion to HEp-2 cells even at the maximum antibody concentration (1:50 dilution) (Figures 7M & N). Taken together, these results suggested that P1-I and P1-IV regions of *M. pneumoniae* P1 protein are surface exposed and are involved in cytadherence.

## Discussion

The human respiratory pathogen *M. pneumoniae* adheres to erythrocytes/respiratory epithelial cells. P1 has been shown to be a major adhesion protein [31-34]. A number of studies using synthetic peptides and monoclonal antibodies against the native P1 protein have illustrated that the P1 epitopes are involved in the adhesion and immune-recognition; however a complete topological mapping of P1-adhesin is still lacking [12,25,27,35]. In the present study, we segmented the entire P1 gene in four regions; P1-I (1069 bp), P1-II (1043 bp), P1-III (1983 bp) & P1-IV (1167 bp) beginning from start residue, ATG and ending with the stop codon. These segments were synthesized by codon optimization replacing all the UGA codons to UGG codons. The P1 fragments were expressed in *E. coli* system and all these fragments were expressed in inclusion bodies. A protocol was developed to purify these protein fragments to near homogeneity and to obtain these proteins in large amount.

The testing of P1 protein fragments with anti-*M. pneumoniae* sera and sera of *M. pneumoniae* infected patients revealed that all these protein fragments were recognized by these sera, thereby suggesting that the immunodominant regions are distributed across the entire length of the protein. These results are in agreement with a number of previous reports that showed the presence of immunodominant regions either in the N-, middle and C-terminal segments of P1 protein [21,23,25,27]. A number of previous reports have shown the presence of immunodominant epitopes usually in the C-terminal of *M. pneumoniae* P1 protein [21,23,36], but few reports also showed immunodominant regions in the middle and extreme N-terminal [25,27]. A comparative summary of these results is presented in additional figure file 4 [see Additional file 4]. However, our’s is the first study that systematically scanned the full P1 protein for their immunodominant and cytadherence.

Since P1 protein is considered to be the major ligand mediating attachment, we next tested the ability of the antibodies raised against the four P1 fragments for adhesion detection, surface exposure and adhesion inhibition assays to identify the cytadherence regions. Previously, a number of studies have identified a few *M. pneumoniae* P1 regions involved in cytadherence. Trypsinization of *M. pneumoniae* P1 protein and ability of various fragments or peptides so generated to block cytadherence provided first evidence for the role of P1 protein in cytadherence [4]. Baseman *et al.*, later showed that the treatment of *M. pneumoniae* with protease blocked its adherence to tracheal explants which was restored when P1 was re-generated [32]. Role of *M. pneumoniae* P1 protein in cytadherence was further substantiated by a study where pre-treatment of *M. pneumoniae* with antiserum directed against the P1 protein blocked its cytadherence to hamster tracheal ring up to 80% [37]. Gerstenecker and Jacobs [11] and Opitz and Jacobs [24], showed the involvement of N-terminal, middle and C-terminal segment of *M. pneumoniae* (P1) as well as *M. genitalium* (MgPa) in cytadherence. Although a number of above mentioned studies have highlighted the role of *M. pneumoniae* P1 protein in cytadherence, however a systematic study spanning the entire length of P1 protein is missing. We performed a systematic analysis of surface exposure and cytadherence region(s) for *M. pneumoniae* P1 protein fragments spanning the entire length. Our results showed that the antibodies against P1-I and P1-IV corresponding to N- and C- terminal regions of P1 protein recognized the surface of adhesive mycoplasmas and blocked their attachment when mycoplasmas were pre-incubated with these antibodies. Based on the result presented here, it can be concluded that the adherence regions are located in the N- terminal and C- terminal regions. Interestingly, Pab (rP1-II) and Pab (rP1-III) antibodies failed to block the cytadherence. The finding of an attachment regions located in the C-terminal part of *M. pneumoniae* P1 protein was consistent with a number of previous studies [11,14,23,24,38,39]. Summary of the various P1 cytadherence mapping regions is presented in additional figure file 5 [see Additional file 5].

## Conclusions

Present study describes a systematic approach to delineate the immunodominant and cytadherent regions across the entire length of *M. pneumoniae* P1 protein. Our results showed that the immunodominant regions are present in several positions across the entire length of the *M. pneumoniae* P1 protein, while the N- terminal and C- terminal regions of the protein are surface exposed and antibodies to these two regions significantly block the adhesion. This data plus data from earlier observations thus confirms the functional significance for *M. pneumoniae* P1 protein in adhesion and immunodiagnosis. These results may have important implications in the development of tools for anti-Mycoplasma drug/vaccine development.

## Methods

### Ethics statement

The protocol of this study was approved by Institutional Animal Ethics Committee (IAEC), AIIMS, New Delhi.

Human blood samples used in this study were received from an already-existing collection approved by the Institution Ethics Committee (IEC), AIIMS, New Delhi.

### *Mycoplasma pneumoniae*, HEp-2 cells and culture conditions

The lyophilized ampoule of *M. pneumoniae* standard strain (M129 strain; National Collection of Type Cultures, London, United Kingdom) was reconstituted in Edward Hayflick medium containing PPLO basal broth that was supplemented with 1% glucose (Difco) as the carbon source and 0.0002% phenol red as the indicator. Tissue culture flasks (Nunc, Roskilde, Denmark) were incubated at 37°C aerobically and inspected daily. An exponential growth phase was indicated by a change in color of the medium from red to orange. Cells were harvested at this stage, washed in phosphate-buffered saline (PBS), centrifuged, and the pellet was stored at −70°C. The organism was confirmed by sub-culturing 0.2 ml of the broth culture on PPLO agar plates (Borosil). Plates were incubated at 37°C in 5% CO_2_ incubator and were examined at 3 day intervals. Colonies were confirmed by Dienes staining and PCR.

The human laryngeal carcinoma cell line, HEp-2 (ATCC, MD, USA), was cultured in TTP tissue-culture flasks (Nunc, Roskilde, Denmark) containing RPMI-1640 medium (Gibco BRL, Grand Island, NY, USA) with 25 mM Hepes-buffer (0.01 M N-2-hydroxyethylpip- erazine-N9-2-ethanesulphonic acid, 0.15 M NaCl, pH 7.2), sodium bicarbonate, fetal calf serum 10%, 200 μg ml^−1^ gentamicin and 2 mM glutamine, pH 7.2. HEp-2 cell was maintained by loosening the cells with PBS containing trypsin 0.25% and EDTA 0.02% and new flask was seeded [14].

### Synthesis and PCR amplification of P1 gene fragments

Entire *M. pneumoniae* M129 P1 gene was synthesized in four fragments; N-terminal P1-I (1069 bp), two middle fragments P1-II (1043 bp) and P1-III (1983 bp), and C-terminal P1-IV (1167 bp) fragments by codon optimization replacing 21 UGA to UGG codons (Entelechon GmbH, Germany). To express these P1 gene fragments, four sets of primers were designed, each having two restriction sites either at 5’end or 3’ end; *Nco*I and *Bam* HI were inserted at 5’ end or *Hind* III and *Sal* I were inserted at 3’ end. Table 1 shows the sequence of each primer. PCR was performed in a 50 μl of reaction mixture containing 1U of Taq polymerase, 1X PCR buffer, 200 μM deoxynucleotide diphosphates, 1.5 mM MgCl_2_, 10 pmol of each primer and template DNA. The reaction conditions were standardized at an initial denaturation of 94°C for 5 min, followed by denaturation at 94°C for 30 sec, annealing at 60°C for 30 sec and extention at 72°C for 1 min for 30 cycles. A final extention was done at 72°C for 5 min. All the four amplified fragments were cloned in pGEM-T easy cloning vector. Cloned fragments were confirmed by restriction digestion and sequencing.

**Table 1:** **Primer sequence used to amplify all four fragments of *M. pneumoniae *** **M129 P1 gene**

**Primers**	**Position (bp)**	**Sequences 5’ to 3’**
F-P1-1	1–21	GGCCATGGGATCCATGCATCAAACCAAAAAAACG
R-P1-1	1051–1069	CCAAGCTTGTCGACCCAAGGAGTTGGTGATCC
F-P1-2	953–974	GGCCATGGGATCCATTAAACGGAGTGAAGAGTCA
R-P1-2	1978–1996	CCAAGCTTGTCGACGTTATTGTGAAAGTAGTA
F-P1-3	1875–1896	GGCCATGGGATCCTTACGCGAAGACCTGCAGCTC
R-P1-3	3840–3858	CCAAGCTTGTCGACCGGCTGGGTACTATGGTC
F-P1-4	3729–3749	GGCCATGGGATCCCTGCACTTGGTGAAACCGAA
R-P1-4	4878–4896	CCAAGCTTGTCGACTGCGGGTTTTTTGGGAGG

The first letter of the primer name denotes the direction of the primer: F forward; R reverse.

### Cloning, expression and purification of P1 gene fragments

For the expression, sub-cloning of the P1 gene fragments was done in *Nco*I and *Hind* III linearised pET28b vector. Ligation mixtures were used to transform BL21(DE3) and transformants were selected on kanamycin (25 μg ml^−1^) plates. Plasmid DNA was extracted from overnight cultures and subjected to restriction digestion to check the inserts. BL21(DE3) cells containing the recombinant plasmids were cultivated in 5 ml of LB broth containing kanamycin at 37°C with shaking (250 rpm) until the optical density (OD) reached 0.4 to 0.6. Protein expression was induced by 1 mM IPTG (isopropyl-β-D-thiogalactopyranoside; Sigma). After 5 h of induction at 37°C, bacterial cells were pelleted by centrifugation and the expression of each protein was analyzed on sodium dodecyl sulfate-polyacrylamide gel electrophoresis (SDS-PAGE) gel.

Sub-cellular localization studies were carried out to analyze the expression of protein fragments in *E. coli* cells. Proteins were found to be expressed in the inclusion bodies. For the preparation of inclusion bodies *E. coli* cells were disrupted by sonication in Tris-buffer (0.05 M Tris, pH 8.0, and 0.3 M NaCl) with 1 min pulses at 1 min intervals 10 times using mini probe (LABSONIC^R^ M, Sartorius Stedim Biotech GmbH, Germany)_._ The soluble and insoluble fractions were separated by centrifugation at 14,000 × g at 4°C for 30 min and were analyzed by SDS-PAGE.

To purify the all four P1 fragments, a protocol developed by Jani *et al.* was followed [40]. Briefly, one liter of *E. coli* culture cells expressing each of the protein fragments was grown and induced with 1 mM IPTG. After the induction, the bacterial pellets were obtained by centrifugation and then suspended in 1/20 volume of sonication buffer; 0.05 M Tris (pH 8.0), 0.3 M NaCl and 1% Triton X-100. The cell suspension was sonicated and the suspension was centrifuged at 14,000 × g for 30 min at 4°C. Pellets were washed 4 times with Tris-buffer without Triton X-100 and resuspended in CAPS (N-cyclohexyl-3-amino propanesulfonic acid, pH 11) buffer containing 1.5% Sarkosin and 0.3 M NaCl. Suspensions were incubated for 30 min at room temperature and were centrifuged at 14,000 × g for 10 min at 4°C. Supernatant of each protein was kept with Ni-NTA^+^ agarose resin with constant shaking for 1 h at 4°C. After binding, each supernatant was packed in four different purification columns and the resin was washed 4 times with CAPS buffer (10% imidazole). Bound proteins were eluted with Tris-buffer (pH 8.0) containing 0.25 M imidazole (Sigma-Aldrich, USA). Each protein fragments were eluted in 5 ml of buffer collecting in ten different fraction of 0.5 ml each. Eluted protein fractions were analyzed on 10% SDS-PAGE gels and fractions containing the recombinant proteins with a high degree of purity were pooled separately. The pooled protein fractions were extensively dialyzed against PBS, pH 8.0 and the protein concentration was determined by Bradford method. The eluted recombinant proteins were denoted as rP1-I, rP1-II, rP1-III and rP1-IV for protein fragments P1-I, P1-II, P1-III and P1-IV respectively.

### SDS-PAGE and western blotting

To analyze the expression of all four recombinant proteins, induced and un-induced *E. coli* pellets from 1 ml of grown cultures were resuspended in 100 μl of 1× SDS sample buffer (62.5 mM Tris–HCl, pH 6.8, 10% glycerol, 2.3% w/v SDS, 5% v/v β-mercaptoethanol and 0.05% w/v bromophenol blue) and boiled for 5 min. The proteins were resolved on 10% SDS-PAGE gel and subsequently stained with Coomassie brilliant blue R-250. To ascertain the expression of the recombinant proteins, western blotting was performed from *E. coli* cell extracts. For immunoblotting, after separating proteins on SDS-PAGE gel, the resolved proteins were transferred onto a nitrocellulose membrane (Sigma-Aldrich, USA) in a trans-blot apparatus (Mini-PROTEAN III, Bio-Rad, USA). The membranes were blocked in blocking buffer (5% skimmed milk in PBS-Tween-20) at room temperature for 2 h. The blots were washed with PBS-Tween-20 (PBS-T) and incubated with monoclonal anti-6XHis primary antibody (Sigma-Aldrich, USA; 1:3,000 dilutions) for 1 h. Blots were subsequently washed and incubated with secondary anti-mouse IgG antibody conjugated with horseradish peroxidase (1:3,000 dilutions). The blots were developed with 3, 3’-diaminobenzidine tetrabenzidine hydrochloride (DAB)-H_2_O_2_ (Sigma-Aldrich, USA)_._

Purified recombinant proteins were analyzed for their reactivity with anti-*M. pneumoniae* antibodies (procured from Public Health Laboratory, London) and sera of *M. pneumoniae* infected patients collected from patients with community-acquired pneumonia who tested positive for IgG antibodies to *M. pneumoniae* (Serion Classic ELISA kit; Serion GmbH, Wurzburg, Germany). The membranes having purified recombinant P1 protein fragments were blocked with 5% skimmed milk in PBST at room temperature for 2 h. After washing with PBST, the blots were incubated with either anti-*M. pneumoniae* IgG antibody (1:3,000 dilutions) or with sera of *M. pneumoniae* infected patient (1:50 dilutions) in two independent experiments. For the negative control, human serum from healthy patient (1:50 dilutions) was used. These blots were washed and then incubated with goat anti-rabbit IgG or goat anti-human IgG antibodies conjugated with horseradish peroxidase (1:5000 dilutions). The blots were subsequently developed with 3, 3’-diaminobenzidine tetrabenzidine hydrochloride (DAB)-H_2_O_2_.

### Immunization of Rabbits for raising antibodies against P1 protein fragments rP1-I, rP1-II, rP1-III and rP1-IV

To characterize the immunogenic potential of recombinant P1 protein fragments, New Zealand white rabbits were used for the immunization with the approval of the Animal Ethics Committee, in accordance with the rules and regulations set forth by the AIIMS Animal Ethics Committee. Immunization was carried out with 6 week old New Zealand white rabbits which were maintained in the animal facility of AIIMS. Before immunization, pre-bleed sera were collected from each of these rabbits. Rabbits were immunized with 200 μg of purified recombinant P1 protein fragments (rP1-I, rP1-II, rP1-III and rP1-IV) emulsified in equal volume (300 μl) of complete Freund’s adjuvant (CFA, Sigma_-_Aldrich, USA) intramuscularly. Rabbits were subsequently boosted with 200 μg of same protein fragments emulsified in equal volume (300 μl) of incomplete Freund’s adjuvant (CFA, Sigma_-_Aldrich, USA) through the same route on the 28^th^ and 56^th^ day. Each one of the control rabbit was immunized with complete or incomplete Freund’s adjuvant in PBS according to the immunization schedule. Blood samples were collected from each of the rabbit by ear vein puncturing on 14, 21, 35, 49 and 63 days. The serum was separated by centrifugation and stored at −20°C for further analysis. The rabbit sera were denoted as Pab (rP1-I), Pab (rP1-II), Pab (rP1-III) and Pab (rP1-IV) respectively. IgG antibody responses against the recombinant protein fragments were analyzed by ELISA and end point titers were determined. To confirm the specificity of antisera, western blot analysis was carried-out for each of the recombinant protein fragments with each antiserum.

### Comparative analysis of recombinant P1 protein fragments by western blotting

In this experiment, equal amount (1 μg) of purified recombinant P1 protein fragments (rP1-I-IV) were run in two separate SDS-PAGE. SDS-PAGE of all the four purified P1 protein fragments was transferred to two separate nitrocellulose membrane to perform western blotting. After blocking with 5% skimmed milk in PBS-T one membrane was then incubated with primary antibody (pooled sera of *M. pneumoniae* infected patients, 1:50) and second membrane was incubated with primary anti-*M. pneumoniae* antibody (1:3,000 dilutions) for 1 h. After washing with PBS-T first membrane was incubated with secondary antibody goat anti-human IgG and second membrane with secondary antibody goat anti-rabbit IgG conjugated with horseradish peroxidase (1:5000 dilutions) for 1 h. The membrane was developed with DAB and H_2_O_2_.

### Reactivity of recombinant P1 protein fragments to patient sera

All the four recombinant P1 protein fragments; rP1-I, rP1-II, rP1-III and rP1-IV were analyzed for their reactivity to twenty five sera of *M. pneumoniae* infected patients and sixteen healthy patient sera using ELISA assay as well as fifteen sera of *M. pneumoniae* infected patients by western blot analysis. Western blot analysis was performed as described above using equal amount of recombinant proteins. For the ELISA analysis, 96-well microplates (Nunc, Roskilde, Denmark) were coated with 50 ng of either of the four P1 protein fragments in 0.06 M carbonate/bicarbonate buffer (pH 9.6) per well. The plates were kept overnight at 4°C and next day the well were washed with PBS-T and blocked with 5% skimmed milk in PBS-T for 2 h at room temperature. The antigen coated wells were next incubated with sera of *M. pneumoniae* infected patients (1:50 dilutions) for 1 h at 37°C. After incubation, plates were washed with PBS-T and incubated with secondary goat anti-human antibody conjugated with horseradish-peroxidase (1:3,000 dilutions) for another 1 h at 37°C. The enzyme reaction was developed by addition of TMB/H_2_O_2_ substrate (Bangalore Genei) and was incubated in dark for 30 min at 37°C. The reaction was stopped with 2 N H_2_SO_4_ and the absorbance was read at 450 nm wavelength using micro-plate ELISA reader (Bio-Tek Microplate Reader, USA).

### *M. pneumoniae* adhesion assay

HEp-2 cells (5×10^4^ HEp-2 cells ml^−1^), in RPMI-1640 medium with penicillin (100 U ml^−1^) 0.05% were added to 24-well Multi-dish plates (Nunc, Roskilde, Denmark) using sterile glass cover slips underneath. The plates were incubated overnight in 5% CO_2_ at 37°C. Next day, HEp-2 cells in each well were infected with the *M. pneumoniae* RPMI-suspension (50 μl well^−1^) and incubated for 6 h in 5% CO_2_ at 37°C. The infected HEp-2 cells were fixed in methanol 100% (1 ml well^−1^) at −20°C for 1 h and washed with PBS. To detect the adhering mycoplasmas, 100 μl per well of each of the primary antibodies anti-*M. pneumoniae* antibodies, Pab(rP1-I), Pab(rP1-II), Pab(rP1-III), or Pab(rP1-IV (1:500 dilutions) were added and were incubated for 1 h at 37°C. Wells were washed subsequently and later 100 μl of secondary fluorescein isothiocyanate (FITC)-conjugated goat anti-rabbit IgG (whole molecule, 1:100 dilutions) (Santa Cruz Biotech, USA) was added. The cells were washed twice in PBS before and after the addition of antibodies. Cells were subsequently incubated with Evans Blue diluted 1:10 for 30 min at 37°C. Finally the cells were washed with double distilled water.

### *M. pneumoniae* adhesion inhibition assay

For the adhesion inhibition assay, protocol developed by Svenstrup *et al.* was followed [14]. Briefly, the *M. pneumoniae* suspension (50 μl) was pre-incubated for 2 h at 37°C with 50 μl of anti-*M. pneumoniae* antibodies, Pab (rP1-I), Pab (rP1-II), Pab (rP1-III) or Pab (rP1-IV) in different dilutions (1:50, 1:100, 1:200 and 1:500) before incubation of the HEp-2 cells. The *M. pneumoniae***-**antibodies suspension (100 μl) was then added to the HEp-2 cells together with 1 ml of RPMI with penicillin and incubated overnight in 5% CO_2_ at 37°C. Fixation and addition of secondary antibodies were carried-out as described in the adhesion of *M. pneumoniae.* To further confirm the adhesion inhibition, the assay was performed as mentioned above except that DAPI was added at the end of the assay for further 30 min at room temperature.

### *M. pneumoniae* surface exposure assay

To detect *M. pneumoniae* surface protein, the primary antibodies were added before methanol fixation. Otherwise, the procedure was the same as described for the *M. pneumoniae* adhesion assay*.*

### Indirect immunofluorescence microscopy (IFM)

Samples prepared for *M. pneumoniae* adhesion assay, *M. pneumoniae* adhesion inhibition assay and *M. pneumoniae* surface exposure assay were analyzed by IFM using Olympus BX51upright fluorescence microscope. Before microscopy analysis, a drop of anti-fade solution (p-phenyldiamine dihydrochloride 1 μg ml^−1^ in PBS 10% and glycerol 90%, pH 9.0) was placed between the glass cover slips and the slides.

## Competing Interests

The author(s) declare that they have no competing interests. Patent application (770/DEL/2012) has been filed under title “Development of immunoassay based on recombinant *Mycoplasma pneumoniae* P1 protein fragments”. This work was funded by Indian Council of Medical Research, New Delhi and has applied for patents.

## Author’s contributions

BK wrote the manuscript and performed the experiment as a part of Ph.D thesis. RC conceived, designed experiments and provide lab facilities and reagents. PM assisted with study design and data interpretation. Both RC and PM edited the manuscript. All authors read and approved the final manuscript.

## Supplementary Material

Additional file 1 **Immune response of P1 protein fragment rP1-I in rabbits.** Bar diagram showing immune responses in four different White New Zealand rabbits immunized with purified recombinant protein fragment, rP1-I with complete/incomplete Freund’s adjuvant. Control rabbits were injected with complete/incomplete Freund’s adjuvant in normal saline according to the immunization schedule. Click here for file

Additional file 2 **Western blot analysis of recombinant P1 protein fragments with rabbits pre-bleed sera.** P1 protein fragments rP1-I, rP1-II, rP1-III & rP1-IV were separated on SDS-PAGE and blots were probed with pre-bleed sera showing no reactivity. Click here for file

Additional file 3 **IFM Adhesion inhibition assay with DAPI staining.***M. pneumoniae* were pre-incubated with monospecific antibodies in different dilutions (1 in 50, 1 in 100, 1 in 200, 1 in 500) before infection of the HEp-2 cells. *M. pneumoniae* infected HEp-2 cells were stained with Evans blue (red) and DAPI (blue). The *M. pneumoniae* microcolonies attached to HEp-2 cells are detected by (a-d) Pab (rP1-I), (f-i) Pab (rP1-IV) and (e & j) pre-bleed rabbit sera with FITC conjugated secondary antibody (green fluorescence). The nuclear material of *M. pneumoniae* microcolonies were not detected by DAPI staining. Click here for file

Additional file 4 **Comparative study of Immunodominant region(s) of P1 protein of *M. pneumoniae*.** Comparison of the immunodominant regions identified in the present study and a number of previous studies. ★ Immunogenic region, aa Amino acid, nt Nucleotide. Click here for file

Additional file 5 **Comparative study of cytadherence region(s) of P1 protein of *M. pneumoniae*.** Comparison of cytadherence regions identified in the present study and a number of previous studies. ★ Cytadherence region, aa Amino acid, nt Nucleotide. Click here for file
