# Supplementary figures and images for: Delineation of immunodominant and cytadherence segment(s) of Mycoplasma pneumoniae P1 gene
Source: BMC Microbiol. 2014 Apr 28;14:108. doi: 10.1186/1471-2180-14-108 (PMC4021176; doi:10.1186/1471-2180-14-108)

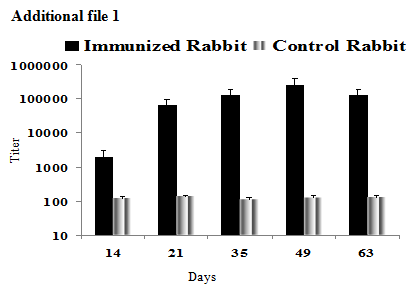

Supplement: Additional file 1 — Immune response of P1 protein fragment rP1-I in rabbits. Bar diagram showing immune responses in four different White New Zealand rabbits immunized with purified recombinant protein fragment, rP1-I with complete/incomplete Freund’s adjuvant. Control rabbits were injected with complete/incomplete Freund’s adjuvant in normal saline according to the immunization schedule. [file 1471-2180-14-108-S1.tiff]

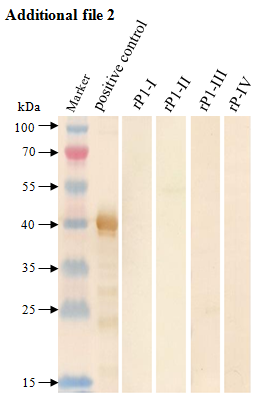

Supplement: Additional file 2 — Western blot analysis of recombinant P1 protein fragments with rabbits pre-bleed sera. P1 protein fragments rP1-I, rP1-II, rP1-III & rP1-IV were separated on SDS-PAGE and blots were probed with pre-bleed sera showing no reactivity. [file 1471-2180-14-108-S2.tiff]

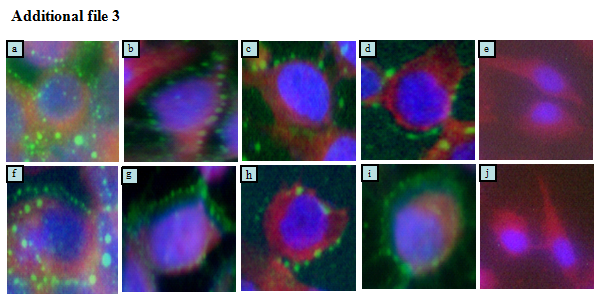

Supplement: Additional file 3 — IFM Adhesion inhibition assay with DAPI staining.M. pneumoniae were pre-incubated with monospecific antibodies in different dilutions (1 in 50, 1 in 100, 1 in 200, 1 in 500) before infection of the HEp-2 cells. M. pneumoniae infected HEp-2 cells were stained with Evans blue (red) and DAPI (blue). The M. pneumoniae microcolonies attached to HEp-2 cells are detected by (a-d) Pab (rP1-I), (f-i) Pab (rP1-IV) and (e & j) pre-bleed rabbit sera with FITC conjugated secondary antibody (green fluorescence). The nuclear material of M. pneumoniae microcolonies were not detected by DAPI staining. [file 1471-2180-14-108-S3.tiff]

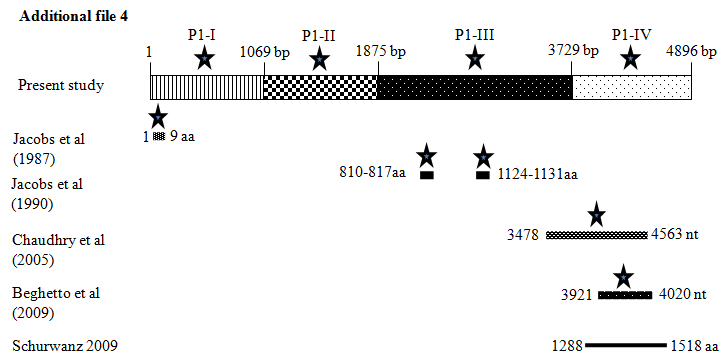

Supplement: Additional file 4 — Comparative study of Immunodominant region(s) of P1 protein of M. pneumoniae. Comparison of the immunodominant regions identified in the present study and a number of previous studies. ★ Immunogenic region, aa Amino acid, nt Nucleotide. [file 1471-2180-14-108-S4.tiff]

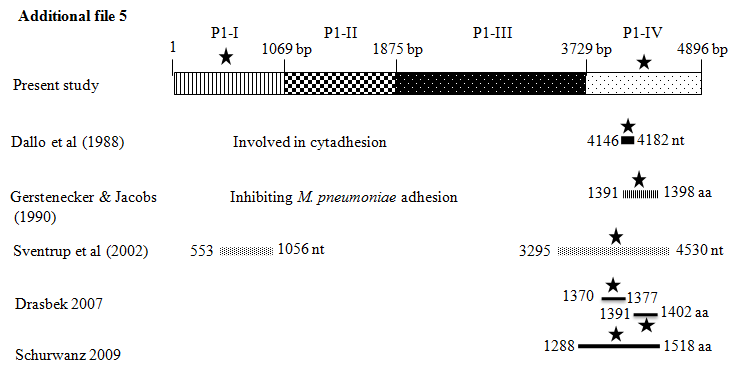

Supplement: Additional file 5 — Comparative study of cytadherence region(s) of P1 protein of M. pneumoniae. Comparison of cytadherence regions identified in the present study and a number of previous studies. ★ Cytadherence region, aa Amino acid, nt Nucleotide. [file 1471-2180-14-108-S5.tiff]
